# Immunological Resistance of *Pseudosuccinea columella* Snails From Cuba to *Fasciola hepatica* (Trematoda) Infection: What We Know and Where We Go on Comparative Molecular and Mechanistic Immunobiology, Ecology and Evolution

**DOI:** 10.3389/fimmu.2022.794186

**Published:** 2022-01-24

**Authors:** Annia Alba, Antonio A. Vázquez, Jorge Sánchez, Benjamin Gourbal

**Affiliations:** ^1^ Centro de Investigaciones, Diagnóstico y Referencia, Instituto “Pedro Kourí” de Medicina Tropical, La Habana, Cuba; ^2^ IHPE, Univ Montpellier, CNRS, IFREMER, Univ Perpignan Via Domitia, Perpignan, France

**Keywords:** host–parasite interaction, snail immunity, cost of resistance, selection of resistance, immuno-ecology

## Abstract

One of the most interesting biological models is that of snail–trematode interactions, many of which ultimately result in the transmission of several important diseases, particularly in the tropics. Herein, we review the scientific advances on a trematode–snail system in which certain populations of *Pseudosuccinea columella* (a common host species for trematodes) have been demonstrated naturally-resistant to *Fasciola hepatica*, in association with an effective encapsulation of the parasite by innate immune cells of the host, the hemocytes. Emphasis is made on the molecular and immunological features characterizing each *P. columella* phenotype in relation to their anti-parasitic competence, their distinctive ecological patterns and the existence of a significant cost of resistance. An integrative overview of the resistance to *F. hepatica* through comparative immunobiology, genetics and ecology is presented to hypothesize on the possible origins and evolution of this phenomenon and to postulate significant roles for parasite mediated-selection and environmental factors in shaping and maintaining the resistant phenotype in the field. Lastly, clues into future experimental perspectives to deeply characterize the interplay between *P. columella* and *F. hepatica* and the immunobiology of the resistance are also included. The advances revised in the present paper are only beginning to unravel mechanisms of anti-parasite innate defense responses and their evolutionary bases, and can facilitate the development of prospective approaches towards practical applications of *P. columella* resistance.

## Introduction

The effects of parasitism on populations of the host are significant: (i) from exerting density-dependent regulation, (ii) actively contributing to the diversity patterns of populations of the host, (iii) affecting their genetic structure through selection, and possibly, (iv) the maintenance of sexual reproduction, to (v) leading in many cases, to co-speciation through host–parasite interactions (1). Concomitantly, the study of host–parasite interactions is particularly essential for the comprehension of the epidemiology and transmission of infectious diseases as there is a reciprocal natural selection between host resistance and parasite infectivity that impacts not only the spatio-temporal dynamics of parasite circulation but also the genetic structure of both entities (2). In this sense, such interplay also contributes to shaping the defense system of the host, with the latter evolving under the influence of two different but equally important selective pressures: to fight back, in a regulated and effective manner, the plethora of infective or harmful agents while remaining tolerant to host self (namely, microbiota).

As parasites continuously evolve to improve their fitness for infecting, surviving, and to be transmitted while thriving at the expense of host resources, a reduction in fitness is expected to occur in parasitized hosts compared to non-parasitized, due to the inherent mechanical, energetic and/or physiological damage exerted by the parasite. This could result in a significant selective pressure as, in turn, hosts must adapt to parasite changes to resist or to tolerate the aggression in an evolutionary “arms race” (3). Nonetheless, examples of host resistance in nature are not plentiful, as these very rare genotypes are usually less abundant due to a fitness cost of such resistance in the absence of parasitism (4). Notwithstanding, a few different types of selection of resistance have been suggested from theoretical studies on the evolution of resistance to parasites [see (5) for review]. Briefly, there is the well-known: i) frequency-dependent selection related to co-evolution of hosts and parasite under the Red Queen principle in which the parasite “tracks” the common hosts genotypes, ultimately reducing their fitness and favoring rare genotypes in a time-lagged parasite-driven genetic selection in host populations. Other theories include: ii) the directional selection for increased resistance or iii) susceptibility, and iv) the stabilizing or v) disruptive selections, all of which are tightly related to the extent of the selective pressure exerted by the parasite and/or to the degree of the associated cost of resistance. The type of selection that occurs varies between particular localities or years and it is postulated that such variation is strongly related to the nature, strength, and shape of the trade-offs associated with resistance, which are highly influenced by ecological factors (5).

The acknowledgment of the impact of environmental variables (e.g., climate, seasonality, other infections, etc.) on host–parasite interactions has been on the rise with several studies accounting for their direct influence on host susceptibility and pathogen dynamics, as environmental changes can alter the encounter and compatibility filters, the immune competence of the host and the immunobiological interplay of the system, and the final outcome of infection [see (6–8) for review]. Furthermore, the indelible link between ecological interactions and evolutionary changes through space and time in host and parasite populations (and their interplay) determines the need for integrative approaches across multiple scales of biological organization (9). This would help to comprehend evolutionary dynamics of disease transmission, to identify general processes and principles within host–parasites interactions that transcend taxonomic boundaries (2), and to attempt the understanding of spatio-temporal variation in ecological factors and their influence on parasite-mediated selection through associated trade-offs for resistance (5).

Within host–parasite systems, snail–trematode models are significant for comparative immunology, evolutionary biology, and health sciences (10). The class Trematoda (Platyhelminthes) groups many parasites highly relevant to public and veterinary health and they present an almost strict dependence upon molluscs, particularly snails acting as intermediate hosts. As a result, snails need to cope with the deleterious effects of infection in an ever-changing environment. As a very interesting model, the interaction of the snail *Pseudosuccinea columella* with the trematode *Fasciola hepatica* has aroused a multi-approach interest integrating disciplines, from classical malacology and parasitology to combined immunological, ecological, and evolutionary perspectives. This snail–trematode system stands out by presenting two different phenotypes within the same species *P. columella* when infection by *F. hepatica* occurs: some populations have susceptible individuals (with a varying degree of compatibility among populations) while others are completely resistant to the parasite. There are studies that show the worldwide distribution of susceptible populations (11) usually displaying variations in compatibility traits [see (12, 13) for discussion]. Such variation, dependant on the genotypes of the infecting parasites, reveals a co-evolution towards a certain degree of genetic matching between the host and the parasite known as polymorphism of compatibility (14, 15). However, resistant populations have been only reported until now in specific natural field sites within the Caribbean island of Cuba and whenever tested, always succeed in fighting back the infection regardless of the *F. hepatica* isolate, the parasitic dose, or even the infection scheme used (e.g., (12, 13, 16). Immunological and molecular studies reflect a constitutively enhanced immune competence of resistant compared to susceptible populations (17, 18), although such selection for resistance is thought to carry a cost in fitness in the absence of parasites; reproductive constraints have been associated to resistant *P. columella* (18–20). In this sense, distinctive ecological patterns have been related to each phenotype; in particular, resistant *P. columella* always occurs in relatively soft/acid waters with low malacological diversity (19).

Herein, we will briefly present this host–parasite system by reviewing the current knowledge and advances made on the topic of *P. columella* resistance from different biological perspectives, namely, phenotypic, molecular, immunological, and ecological standpoints. Afterwards, we will discuss the possible origins of the resistant phenotype based on the preliminary evidences and on theoretical understanding of parasite and environmental-driven selection. Lastly, as much remains to be studied for a proper understanding of the mechanistic and evolutionary bases featuring the resistant/susceptible phenotypes, clues into some future experimental perspectives will be briefly stated. The advances revised herein are beginning to unravel mechanisms of anti-parasite innate defense responses and their evolutionary bases, and can facilitate the development of prospective approaches towards practical applications of *P. columella* resistance.

## Resistance of *P. columella* to *F. hepatica* in Cuba: What We Know

Cuba, the biggest archipelago of the Caribbean basin, is mainly plains and floodable lowlands under a tropical regime of warm temperatures and frequent rainfall throughout the year, all of which favor the occurrence of the two lymnaeid snails *Galba cubensis* and *P. columella* (21). Both species act as intermediate hosts of a number of parasites, namely, the medically and veterinary relevant trematode *F. hepatica* (19, 22, 23), the main etiological agent of the (re-)emergent significant zoonosis known as fasciolosis [see (24)]. In this sense, elevated rates of transmission of *F. hepatica* in nature (mainly related to human activities such as cattle husbandry) are believed to occur all year-round (22, 25) supported by high prevalence of infection in both lymnaeid snail species reported from field (19, 22, 26) and laboratory studies [e.g., (12, 13)]. Although ecological and parasitological studies point at *G. cubensis* as the main intermediate host of *F. hepatica* in Cuba and in the Caribbean (13, 21, 22, 27), *P. columella* is also a worldwide-recognized host of the parasite (11). In Cuba, *P. columella* presents a wide distribution in the western and central regions of the country (19). Moreover, high infection rates of *P. columella* by *F. hepatica* from 1 to 10% (19, 23), and even up to 100% prevalence (13) have been recorded respectively from the field and from experimental studies in Cuba.

**Figure 1:**
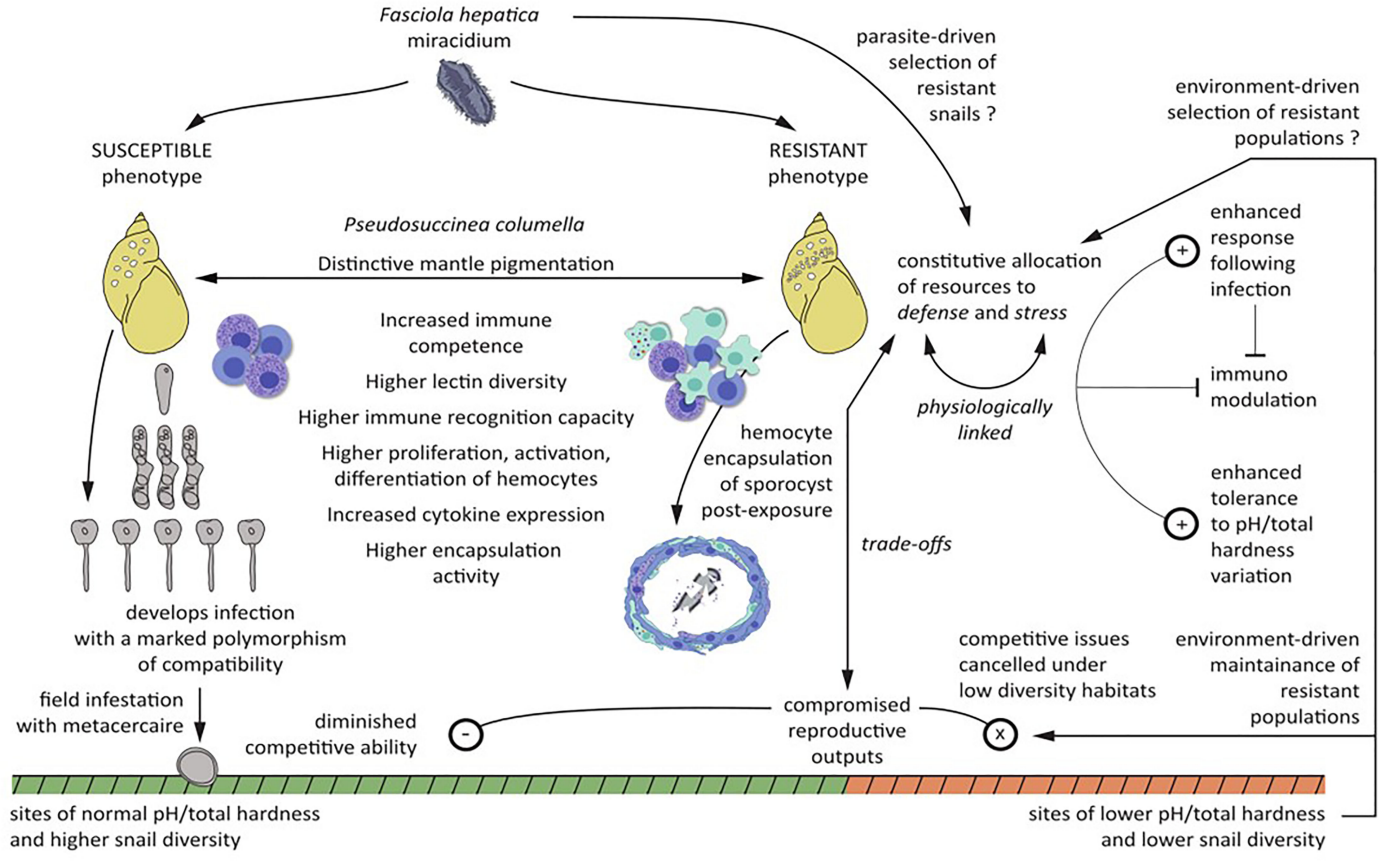
Comprehensive view of the main phenotypic features of resistant and susceptible *Pseudosuccinea columella* to *Fasciola hepatica* and the main results and hypothesis derived from comparative molecular and mechanistic immunobiological, ecological, and evolutionary approaches applied to this system. Symbols in circles refers to enhanced (+), diminished (−), and canceled (x) pathways.

However, one of the most interesting facts related to *P. columella* in Cuba is the occurrence of, at least six field populations that are naturally resistant to *F. hepatica* infection. The six populations occur in the localities of La Playita, Loma Candelaria, La Palma, El Azufre, La Coca, and Babiney, with the latter being the only one found in central Cuba [see (19) for details on their distribution]. A distinctive morphological feature consisting of a belt-like shape of small, highly abundant sharp-whitish spots in the central region of the mantle with wider and sparser spots in the upper and lowers sides has been associated to these populations [see (18, 28), and **Figure 1**]. All *P. columella* snails displaying such phenotypic markers have never been found infected with *F. hepatica*, either in nature or after experimental challenges, regardless of the isolates of the parasite used, the parasite dose or the infection scheme (12, 13, 16, 20, 28). In this sense, histological sections of exposed resistant *P. columella* showed that the penetration of *F. hepatica* miracidium into the snail is rapidly followed by an effective encapsulation of the parasite larvae by the hemocytes of the host (circulating immune cells of snails), observed at 24 h post-exposure (28). This powerful immunological reaction is believed to be at the base of the resistance of *P. columella* to *F. hepatica* (17, 28).

Although resistant and susceptible *P. columella* belongs to the same species accordingly to significant anatomical, conchiological and genetic data (18, 29), nuclear polymorphic genetic markers and mitochondrial haplotypes cluster resistant populations in a different clade from susceptible snails, with little within-group polymorphism (11, 16, 19). This segregation implies a certain genetic determinism of resistant *P. columella* and opens to the possibility that such phenomenon was selected from or has derived in a different genetic pool of the majority of susceptible *P. columella* populations worldwide (11, 19). Furthermore, resistant populations, particularly those from La Coca and Babiney, showed an overall higher allelic richness and higher genotypic diversity than susceptible populations, and they not only significantly differed from susceptible populations but also between themselves, possibly as a result of different introductions between western and central regions of Cuba and to their *in situ* evolution (19).

### Comparative Molecular and Immunobiological Studies Between *P. columella* Phenotypes: Evidences of Increased Immune Competence Towards *F. hepatica* Associated With the Resistant Phenotype

For two decades, several efforts have been made to challenge the resistant phenotype by increasing the odds for *F. hepatica* success. In this sense, resistant *P. columella* snails have been exposed to different sympatric and allopatric *F. hepatica* from Cuba [e.g., (13, 16)], which are usually genetically diversified [see (25) for details]. In addition, they have been also confronted against isolates of *F. hepatica* from different countries (Dominican Republic—narrow geographic scale and France—large geographic scale), trickle infections (3 exposures every 3 days) and single infections with increasing parasite loads (12). Although these attempts aimed at increasing the probability of encounter of compatible genotypic host–parasite individual combinations and at potentially decreasing the effectivity of the immune response of the host (such effects have been observed in susceptible populations used as control), they have all failed in rendering phenotypically resistant snails infected. Such stability and universality (at least at the above mentioned scale) of this phenotype paired to the potent immune response associated to it (17, 28), point at the occurrence of an immunological resistance that is not restricted by specific interactions of matching genotypes between the host and the parasite. It is, therefore, rather a phenomenon of immunological resistance than one of incompatibility and that, noteworthy, has been selected in nature (18, 28). We have postulated that a major factor/function associated to a single locus or a few loci is present or highly abundant in the genetic clade of resistant *P. columella* snails. Such feature is believed to be involved proximately or ultimately, in the immunobiological interaction with *F. hepatica*, interfering with biologically relevant functions of the parasite coded by a single locus or a few loci highly conserved (17). Contrastingly, given the polymorphism of compatibility resulted from exposing different susceptible snails to different parasite isolates [see (12, 13) for discussion], it was speculated that the major factor highly present in resistant snails is absent/scarce in susceptible *P. columella* and, consequently, it is the interplay among several other factors/functions between the susceptible host and the parasite that dictates the final outcome and the intensity of the infection, possibly preferentially following a matching allele model (17).

In this sense, the comprehension of the molecular and mechanistic bases that mediate *P. columella* resistance to *F. hepatica* is pivotal for comparative immunology and evolutionary biology. The rapid orchestration of the protective cellular immune response against *F. hepatica* in the resistant phenotype during the first 24 h post-infection (28) supports the notion of the occurrence of at least some constitutive elements participating in the resistance (18). In fact, hemocytes from naive (non-exposed) resistant snails significantly associate to higher encapsulation activity of *F. hepatica* sporocysts *in vitro*, compared to immune cells from naive susceptible snails (17). With all this in mind, the whole-snail transcriptome of *P. columella* and the differential proteome of the albumen gland (organ involved in both reproduction and immune response) prior infection (naïve conditions) were analyzed to compare the constitutive “molecular base line” between resistant and susceptible phenotypes (18). From this study, significant enrichments of five functions/biological processes in the resistant phenotype were recorded, among which the immune/stress response was particularly noted. Such enrichment in these biological functions was supported by the significant abundance of different transcripts identified as orthologues of recognized immune molecules involved in the recognition of pathogens, in immune regulation, and defense activation/response in association to resistant *P. columella*. In this sense, several types of lectins and pattern recognition receptors, CD109, cytokines and cytokine related molecules [e.g., macrophage migration inhibitory factor (MIF), granulocyte colony stimulatory factor receptor (G-CSFR), transforming growth factor 1-beta (TGF1β)], and also of signaling/regulatory transcripts (e.g., Toll-like receptors (TLR), protein C kinase (PKC), members of the superfamily of tumoral necrosis factor (TNF) receptors, interferon regulatory factors (IRF) and molecules with leucine rich repeats), and enzymatic antioxidants and molecular effectors such as ferritine, nitrite oxide synthase, catalase and superoxide dismutase where found overexpressed in resistant versus susceptible snails (18). Orthologues of these transcripts have been recognized as relevant for the immune response in other mollusk species such as *Lymnaea stagnalis* and *Biomphalaria glabrata*, and particularly, in their defense against trematodes [e.g., (30–35)]. In addition, other significant immune effectors, i.e., a G-type lysozyme and a lipopolysaccharide-binding protein/bactericidal permeability-increasing protein (LBP/BPI) (36, 37), were also revealed from the analysis of the albumen gland to be only present in the proteome of resistant snails (18). The latter made patent, at the molecular level, the significant and constitutive allocation of resources towards self-maintenance, which possibly occur at the expenses of other competing biological functions [e.g., reproduction; (38)] and thus, implying possible trade-offs associated to resistance.

In terms of *P. columella*–*F. hepatica* interaction, apparently, mannosylated residues in the surface of *F. hepatica* sporocysts and mannose recognition factors have central roles in the molecular interplay between the parasitic larvae and the snail hemocytes. Indeed, a marked inhibition of parasite encapsulation by *P. columella* hemocytes was observed when the cells were pre-incubated with mannose, irrespective of the phenotype (17). Among the previously mentioned overrepresented molecules associated with resistant *P. columella*, the high abundance of different types of lectins and in particular, those involved in mannose recognition, e.g., mannose-binding protein C, C-type mannose receptor 2, mannose binding protein, paired with the overexpression of immune-related signaling molecules (18) might be fundamental in driving a protective response in the resistant phenotype that begins by the proper recognition of *F. hepatica*. At the functional level, qualitative and quantitative differences in the surface of hemocytes between phenotypes can be presumed from the different degrees of inhibition of the encapsulation of *F. hepatica* by *P. columella* hemocytes recorded when the cells were pre-incubated with a variety of carbohydrates [i.e., glucose, galactose, fructose; (17)].

Other functional differences were observed when different aspects of the immune response were studied and compared between *P. columella* phenotypes following experimental infection. A significant increase of the number of circulating hemocytes, of the proliferative activity of blast-like hemocytes and in the adherence and spreading capacity of large hemocytes were verified to occur in resistant snails, at 24 h post-exposure to *F. hepatica*. Notably, the highest *in vitro* encapsulation activity, resulting in the total formation of a hemocyte capsule around *F. hepatica* sporocyst was recorded only in exposed resistant snails, at 24 h post-exposure (17). Additionally, a rapid increase of the expression of the cytokine granuline was recorded at 6 h post-exposure only in resistant snails (17). This cytokine participates in the proliferation, activation, and differentiation of hemocytes in trematode-challenged snails and increased levels have been associated to a decrease of the susceptibility to the infection in *B. glabrata*–*Schistosoma mansoni* system (39). Therefore, not only do resistant snails present a more competent molecular and functional base line prior infection, but they also respond more rapidly and efficiently to the parasite following infection (17, 18). These features endorse the potent immune phenotype that develops in these snails towards *F. hepatica* and indicate that constitutive and inducible elements share roles in the orchestration and amplification of the protective immune response that occurs in resistant *P. columella* (17).

Contrastingly, exposure to *F. hepatica* of susceptible snails rendered a less competent immune phenotype not only compared to resistant snails but also to their naïve conditions. No significant increase, neither of the total hemocytes counts nor of blast-like cells proliferation, were observed in exposed-susceptible snails. In addition, large hemocytes displaying less and/or shorter pseudopods without clear tendency to cohesion or aggregation at 24 h post-exposure compared to naïve conditions were noted, which possibly relates to the diminishment of the encapsulation capacity of *F. hepatica* larvae also recorded at that time point. Furthermore, a significant decrease of granulin expression at 12 h and 24 h time points, below constitutive levels, observed in the susceptible phenotype accounted for an almost 3-fold differential increased expression in resistant compared to susceptible snails at 24 h post-exposure (17). From these results, we suggested that infection by *F. hepatica* involves immunomodulatory/immunosuppressive parasite-driven effects that are rapidly put in place and that only affect the susceptible phenotype, possibly resulting in infection when compatible combinations take place. It has been hypothesized that the constitutive allocation of resources to defence observed in resistant snails contributes to speedily impair such parasite-mediated immunomodulation or that *F. hepatica* immunosuppressive factors are inefficient against resistant snails and, concomitantly, trigger the powerful protective response that develops following *F. hepatica* penetration [see (17) for discussion].

Selection for resistance is often associated with fitness costs, thus understanding how resistant phenotype could maintained in the field is an intricate question. To answer this question it is important to consider the environmental conditions in which host and parasite species evolved. This is particularly true for resistant *P. columella* that always occurs in relatively soft/acid waters with low malacological diversity. The links between resistance, fitness cost and environmental factors need thus to be investigated in the *P. columella*/*F. hepatica* interaction.

### Ecological Patterns Associated With the Resistant Phenotype: Evidences of a Cost for Resistance and Environmental Abiotic Factor Selective Pressures

Taking into account the heavy investment on immune or stress response described in P. columella (see section Comparative Molecular and Immunobiological Studies Between P. columella Phenotypes: Evidences of Increased Immune Competence Towards F. hepatica Associated With the Resistant Phenotype), particularly in naïve conditions, one could ask if there is an ecological cost of P. columella resistance to F. hepatica in the absence of parasitism?

Results from Alba et al. (19) indicate that from all the malacological records of *P. columella* in Cuba, the majority (more than 90% of all field records) belongs to the susceptible phenotype. This contrasts with the supposition that resistant populations are the result of more ancient introductions in the island given its high genetic diversity in relation to the mostly monomorphic nature of susceptible *P. columella* snails from Cuba. This is particularly interesting knowing the diminished competitive potential of resistant snails compared to susceptible snails, resistance seems to impair their apparent capacity to invade and successfully colonize new habitats. In this sense, the analysis of the ecological factors occurring in the different sites where each *P. columella* phenotype exists has rendered a peculiar association: resistant *P. columella* populations were always present in sites of slightly acidic (pH = 6.2 ± 0.12), softer water (total hardness, TH = 6.3 ± 1.03°d) and lower malacological diversity [3.2 ± 1.02 freshwater snail richness (19)]. Extensive ecological studies performed state that most freshwater sites in Cuba are featured by higher pH/TH values [ranging from 7–8.5 to 12–18°d; (40)], and in fact, resistant populations represent only the 1.2% of all the registered accounts of freshwater snail populations in Cuba (19).

To clarify the significance of pH/TH on the occurrence of resistance and susceptible *P. columella*, life-history traits experiments were performed in the laboratory simulating the differential values recorded in the field in association with each phenotype [i.e., 5.9/4°d or 7.8/14°d; (19)]. According to this study, survival and fertility traits of *P. columella* snails are negatively affected by low pH/TH irrespective of the phenotype whereas, overall, all populations performed better at common pH/TH values (similar to those corresponding to the finding of susceptible populations in the field). Nonetheless, resistant populations showed a diminished reproductive potential featured by a lower fecundity rate and late reproductive peaks (La Coca population) or by delayed egg hatching (La Palma population) when compared to susceptible *P. columella*, regardless of the experimental setting (19). Previous studies also reported lower fecundity rates for resistant (La Palma population) compared to susceptible *P. columella* (20). All these results suggest a possible fitness and physiological cost associated with the resistant phenotype of *P. columella* (19, 20).

Interestingly, resistant snails showed higher tolerance to the low pH/TH setting than susceptible snails. Resistant snails presented higher survival, life expectancy at birth and proportion of viable eggs compared to susceptible snails (19). These results suggest that the restricted distribution of the resistant phenotype in low pH/TH water is due to the conjunction of their higher tolerance to these stringent environmental conditions and the compensation of their diminished reproductive and competitive abilities in sites of low malacological diversity (19). It has been reported, under experimental conditions, that the presence of susceptible *P. columella* when rearing resistant snails results in a decrease of the net reproductive rates of the latter. Contrastingly, susceptible snails increase both growth and net reproductive rates when paired with resistant snails (41), which could be related to an unbalanced competition in favor of susceptible snails. In nature, a temporal invasion of a traditionally susceptible *P. columella*-occurring site by a nearby resistant population coincide with the decrease of pH/TH values from 7.5 to 6.5 (19). Furthermore, ecological follow-up studies of temporal dynamics on El Azufre and La Coca (sites of low pH/TH) have shown that resistant *P. columella* occurs always as the more stable population compared to the other mollusk species, even compared to the acknowledged highly invasive gastropods *Tarebia granifera* and *Physa acuta* [see (19, 42)]. Both species are widely distributed in Cuba, and particularly *T. granifera*, has been pointed as strong competitor for pulmonates including lymnaeids.

It is significant to point out that results from the molecular studies are in harmony with what have been observed from the ecological approaches. The molecular base line of resistant *P. columella* is characterized by a significant allocation of resources to the constitutive production of molecules involved in immune defense and stress responses, which could not only endorse the enhanced capacity to react to *F. hepatica* infection but also to abiotic stressors such as particular environmental conditions (18). In this sense, and concordantly with the higher tolerance displayed for pH/TH variations, several transcripts, particularly involved in the regulation of osmotic and pH variations of the internal milieu (43, 44), e.g., several carbonic anhydrase isoforms, and major ion transporters such as Na^+^/K^+^-ATPase, H^+^-ATPase, Na^+^/H^+^-exchanger, are constitutively overexpressed in resistant compared to susceptible snails [see (18)]. This constitutive over-expression of genes involved in adaptation to acidic water in the resistant phenotype may specifically trade-off against reproduction. Concomitantly, resistant snails trigger a powerful protective immune cellular response involving the production of immune effectors following *F. hepatica* penetration (17), resulting also in a potential reproduction trade-off. This is particularly evidenced by the comparative proteomic approach conducted on the albumen gland. Albumen gland guarantees the nourishment of embryos through the production of different elements of the perivitelline fluid (45, 46), but is also involved in production of immune effectors. Thus, the metabolic rearrangements favoring the production of particular immune effectors, regulatory and catabolic enzymes in detriment of overall protein synthesis in albumen gland could be at the base of the reproductive constraints associated to the resistant phenotype (18).

## Possible Influence of Parasite vs. Environmental Factors: Discussing the Selection of Resistance

As previously mentioned, resistant *P. columella* populations could tolerate slightly acid and soft waters better than susceptible snails and they experienced certain reproduction constraints compared to susceptible populations (19, 20). Interestingly, both, a protective immune response towards *F. hepatica* and an enhanced tolerance to pH/TH variations, involved higher molecular and phenotypical fitness to respond to stressors (biotic or abiotic), whereas the impairment of reproductive traits is likely a consequence of energetic trade‐offs among potentially competing physiological processes [i.e., self-maintenance/survival *vs*. reproduction; (38)]. All these could explain the limited distribution of resistant *P. columella* in the field to localities where being more tolerant to variations of water acidity/hardness or more immunologically resistant could be considered as an advantage (i.e., sites with low pH/TH or high *F. hepatica* pressure) thus, compensating for their low reproductive or competitive capacities.

However, the question of the origins of the primary feature of resistant snails (i.e., the immunological resistance to *F. hepatica*) and its association with the higher tolerance to pH/TH variations remain. Noteworthy, both the immune response and acid–base/osmotic regulation, are physiologically linked through several molecular functions, effectors and signaling pathways (47, 48). Changes on environmental and internal pH could lead to an improved immunological condition through an increase of different defense-related traits as observed in other invertebrates, e.g., increase haematopoiesis (49), phagocytic capacities of the hemocytes (48, 49), and acid-balance and anti-oxidative regulatory potential (43). The boosting of such features with pivotal roles in processes such as immune surveillance and immune response [see (50–53) for some examples], could make the snails more prone to efficiently respond to infectious challenges by being “better prepared” to recognize, phagocyte/encapsulate and kill intruders. On the other hand, an enhanced immune responsiveness may involve a metabolic reconfiguration towards a relative increase of pathways related to energy and metabolite production; e.g., respiratory rate, glycolysis, proteolysis and/or lipolysis (54). This could lead to a concomitant increase of protons, reactive oxygen species and CO_2_ levels in the internal milieu, a biochemical setting that it is, then, coped through an enrichment of other related functions, such as antioxidant enzymes, carbonic anhydrase, and certain ion transporters (55, 56). Therefore, the enhanced tolerance to pH/TH variations associated to resistant snails could be a result of such a metabolic reconfiguration as, in aquatic species, the acid–base and osmotic balances are closely linked through the coupled movement of H^+^ and HCO3^−^ with that of Na^+^ *via* ion transporters (44). In this sense, an overexpression of immune-related transcripts/proteins with pro-activating and pro-inflammatory roles, an enrichment of energetic metabolic process, and of carbonic anhydrase and major ion regulators featured the constitutive molecular base line of the resistant phenotype (18).

Unfortunately, all these experimental and theoretical outlines remain inconclusive and from them, the hypothesis on the selection of the primary feature associated to resistance could go either way: is it the higher pH/TH tolerance a “by-product” resulting from parasite-driven selection of resistance, or is it the resistant phenotype a merely “side effect” of an environmental-driven selection for an increased fitness for compensating osmotic and acid–base variations? An environmental-driven selection could, perhaps, easily explain the broad resistance towards *F. hepatica*, irrespective of the infective isolate [see (12, 13, 16)], through an overall enhancement of multiple and “universal” immune functions in the snails. However, from such an environmentally-linked immune reactiveness not selected from specific parasitic pressures, it might be also expected for it to remain fairly “constant” and not to strongly develop into a highly competent induced defence following a particular infection, and possibly, for it to be extended to other infectious challenges. Contrastingly, evidences of a potent and significantly different induced immune response following exposure to *F. hepatica* in terms of hemocyte morphology, proliferation and encapsulation activities, and of granulin expression are reported from resistant but not from susceptible *P. columella* snails. Contrastingly, resistant and susceptible *P. columella* were equally sensitive to experimental infection by *Trichobilharzia* sp. from which high infection rates and cercarial outputs were attained [see (57)]. Moreover, it is noteworthy that resistant snails from La Coca have been found naturally infected with rediae, cercariae and metacercariae of Echinostomatidae (author’s unpublished data). These results suggest that there is a certain specificity of such “enhanced immune competence” towards *F. hepatica* that allows to uniquely recognize and to “better” respond to this particular parasitic challenge and thus, possibly fitting better into a parasite-driven selection hypothesis.

However, we have to be careful to not oversimplify the situation, considering exclusively a simple dichotomy, without considering that both selection favoring resistance and selection favoring pH tolerance could be involved in the observed natural resistant phenotype. To present an integrative view of all these results and of the possible influence of both the parasite and the environment, we hypothesized that a parasite-driven selection took place resulting in a phenotype featured by an immunological resistance to *F. hepatica* and a high tolerance to pH/TH changes that traded-off against reproduction. Frequency dependent selection and/or disruptive selection on resistance associate evolution of resistance through parasite mediated-selection with the occurrence of a fitness cost and of an increase of the genetic diversity of host populations [see (58, 59) for examples], could have occurred in the case of resistant *P. columella* [see (11, 19, 20) for genetic diversity and cost of resistance in *P. columella*)]. While it should be kept in mind that evolutionary processes are time-lagged, it might be argued that impaired competitive capacities due to the trade-off against reproduction could have probably already determined a negative selection for the resistant but less ecologically-competitive phenotype with the diminishment of the parasitic pressure that followed the primary selection for resistance. However, it should be considered that resistant *P. columella* occurred mostly in western Cuba (19), a region that has been recognized for decades to be one of the most heavily impacted by fasciolosis (19, 22, 25, 60, 61). In particular, four resistant populations are even located within or in close proximity to localities where specific transmission foci of *F. hepatica* have been described [i.e., La Palma, El Azufre, La Coca, Loma de Candelaria; see (20, 42, 62, 63)]. Furthermore, high prevalence of infection in lymnaeid snails (19, 22) and in livestock (25, 64), and even human cases and outbreaks (61, 65) have been historically reported in central and western Cuba, all of which presume high odds for host–parasite encounters all year-round. Snail–trematode encountering rate has proved to be significant in the field, even in sites showing low prevalence of patient infections, by the use for example of sentinel snails for *Bulinus globosus*/*Schistosoma haematobium* interaction (66). Concomitantly, experimental and observational evidences endorse a significant role for the environment, maybe in the primary selection but, in any case, in the maintenance of the phenotype in the field, as the trade-offs associated with *P. columella* resistance are compensated by their higher tolerance to the more restrained environmental conditions for other competitor snails that comes with lower pH/TH values. This could be particularly significant if a disruptive or even a directional selection of resistance took place, as the fitness cost associated with *P. columella* resistance is unlikely to be relatively weak given the limited distribution of these populations in nature in spite of their ancient presence in the island.

In any case, while resistant populations have been found so far in Cuba, it might be considered that the primary features of these populations could have been selected somewhere in North America from where *P. columella* is suggested to have originated. However, no exhaustive sampling towards tracking this resistant phenotype has ever been carried out in this region.

**Figure 1** aimed at providing an integrative synthesis of the main phenotypic features of resistant and susceptible *P. columella* to *F. hepatica* and the main results and hypothesis derived from comparative molecular and mechanistic immunobiological, ecological, and evolutionary approaches applied to this system.

## Future Experimental Perspectives: *Where Do We Go From Here*

There is a growing interest for understanding immunobiological interactions within vector snails as they can increase our comprehension of innate defenses from mechanistic and evolutionary points of view, of the dynamic of parasite transmission and of the multiple factors influencing parasite circulation. In the case of *P. columella*–*F. hepatica*, future studies aiming at deciphering the particular immune pathways and molecules involved during resistant/susceptible host–parasite interactions are essential to shed light on the differential determinants mediating each phenotype and to propose global mechanisms of resistance/infection for this system. Multiple “omics” approaches coupled with functional validation (RNA interference: siRNA, dsRNA) of the identified pathways following parasite exposure need to be applied to decipher the molecular and metabolic scenario in susceptible and resistant snails. In this sense, it will be also interesting to establish the inheritance pattern of the identified specific markers through mating experiments, for theoretical and practical uses. However, as self-crossing constitutes the almost exclusive reproductive strategy of *P. columella* (11, 19), it is likely that directed castration should be put in place to resolve this issue.

Due to the association of the resistant phenotype with an increase molecular and phenotypical competence to cope with stress (18, 19), experimental infections on stressed-snails could be worth testing in this model. Environmental factors such as an increase of temperature and starvation are known to affect snail–parasite interaction by decreasing immune capabilities of the host (67, 68), increasing their susceptibility to digenean infections (69) and, even reverting certain resistant phenotypes (70, 71). In this resistant *P. columella* model, the exposure to heat waves, low pH/TH, and low food income prior infection could negatively affect the energetic budget necessary for the sustainability of its enhanced immune competence following *F. hepatica* exposure. Meanwhile, it would be worth exploring if housing susceptible snails in low pH/TH conditions could decrease their sensitivity to *F. hepatica* as it could strengthen the argument towards an environmental-mediated resistance to the parasite. Furthermore, experimental studies assessing life traits and competing capacities of resistant populations reared in sympatry with other freshwater snail species at common pH/TH conditions could gauge the cost of resistance against the most frequently found gastropods in Cuba.

An interesting interaction to approach in the future in this model would be the possibility of *P. columella* of actively avoiding *F. hepatica* infection. Recent studies depicting a scenario referred to as ‘a landscape of disgust’ explain how parasite avoidance shapes the ecology and evolution of host-parasite interactions (72). Heavily miracidia-infested areas in portions of ponds, rivers or other freshwater bodies may prevent *P. columella* of settling, and remain in distal regions with slightly different physical conditions. This, however, would be an interesting fact to explore given that snails usually become infected passively and have little to none chance of avoidance by a direct change in behavior.

Furthermore, field and experimental measures to characterize the encounter filter of resistant *P. columella* (e.g., encounter rates with F. hepatica, attractiveness of *F. hepatica* miracidia) could be also a motivating research with implications not only for biological sciences but also for epidemiology.

In addition, experimental evolution over few generations of parasite resistance on susceptible *P. columella* by selectively pressing with *F. hepatica* or with low pH/TH housing conditions might shed light on the mechanistic bases behind the phenotypic features recorded in resistant populations. It could be also interesting to evaluate the sensibility of resistant and susceptible *P. columella* populations to other trematode parasites, namely, those phylogenetically close to *F. hepatica* and compatible with *P. columella* (e.g., *Fasciola gigantica* and *Fascioloides magna*) to deeply characterize the specificity of the resistance. All of these approaches deserve further investigation.

## Author Contributions

Investigation, AA, AV, and BG. Writing—Original Draft Preparation, AA, AV, and BG. Writing—Review & Editing, AA, AV, JS, and BG. Project Administration, AA and BG. Funding Acquisition, AA, AV, and BG. All authors contributed to the article and approved the submitted version.

## Funding

Partial financial support for this investigation was provided by the subventions granted to AA by the French Embassy in Cuba and to AV by the Institut de Recherche pour le Développement (BEST grant). This study is set within the framework of the “Laboratoires d’Excellences (LABEX)” TULIP (ANR‐10‐LABX‐41). BG was supported by the ANR JCJC INVIMORY (number ANR 13-JSV7-0009) from the French National Research Agency (ANR).

## Conflict of Interest

The authors declare that the research was conducted in the absence of any commercial or financial relationships that could be construed as a potential conflict of interest.

## Publisher’s Note

All claims expressed in this article are solely those of the authors and do not necessarily represent those of their affiliated organizations, or those of the publisher, the editors and the reviewers. Any product that may be evaluated in this article, or claim that may be made by its manufacturer, is not guaranteed or endorsed by the publisher.
